# 4-octyl itaconate reduces human NLRP3 inflammasome constitutive activation with the cryopyrin-associated periodic syndrome p.R262W, p.D305N and p.T350M variants

**DOI:** 10.1007/s00018-025-05699-5

**Published:** 2025-05-23

**Authors:** Cristina Molina-Lopez, Laura Hurtado-Navarro, Luke A. J. O’Neill, Pablo Pelegrin

**Affiliations:** 1 Molecular Inflammation Group, Biomedical Research Institute of Murcia (IMIB), Edificio LAIB 4ª Planta, Carretera Buenavista S/N, 30120 El Palmar, Murcia Spain; 2 School of Biochemistry and Immunology, Trinity Biomedical Sciences Institute, Trinity College Dublin, Dublin, Ireland; 3 Department of Biochemistry and Molecular Biology B and Immunology, Faculty of Medicine, University of Murcia, 30120 Murcia, Spain; 4 CABIMER, Seville, Spain; 5 IdiPaz, Madrid, Spain

**Keywords:** Itaconate, Autoinflammatory disease, CAPS, NLRP3, Inflammasome

## Abstract

Cryopyrin-associated periodic syndrome (CAPS) is a condition characterized by dominant genetic variants in the *NLRP3* gene, which lead to the formation of constitutively active inflammasomes. These inflammasomes play a crucial role in CAPS patients’ inflammatory episodes, these being primarily driven by the production of interleukin (IL)-1b. Although treatment with IL-1 blockers is effective for CAPS, some patients develop refractory responses and adverse reactions to these therapies. Consequently, there is a need for novel treatments for CAPS patients. Promising candidates are the derivatives of itaconate, which have been shown to impair NLRP3 inflammasome activation and IL-1β release in blood mononuclear cells from CAPS patients. In this study, we provide insight into the inhibitory mechanisms by which the itaconate derivative 4-octyl itaconate (4-OI) acts on NLRP3 that has different gain-of-function mutations (p.R262W, p.D305N and p.T350M) associated with CAPS. Notably, 4-OI effectively blocks the basal auto-activation of the inflammasome formed by NLRP3 p.R262W, p.D305N and p.T350M variants, which in turn reduces caspase-1 activation, gasdermin D processing, and IL-18 release. Furthermore, after lipopolysaccharide priming of macrophages, 4-OI also decreases IL-1β gene expression and release. Overall, 4-OI impairs CAPS-associated inflammasome function at multiple levels, meaning that therapeutic agents based on itaconate could be a promising therapeutic approach to managing inflammatory episodes in CAPS patients carrying p.R262W, p.D305N or p.T350M variants.

**Supplementary Information:**

The online version contains supplementary material available at 10.1007/s00018-025-05699-5.

## Introduction

The NLRP3 inflammasome plays a crucial role in the initiation and maintenance of the inflammatory response following tissue damage and infection. In myeloid cells, NLRP3 is activated in response to various damage–associated molecular patterns (DAMPs). These DAMPs can disrupt the plasma membrane, destabilize lysosomes, and produce mitochondrial reactive oxygen species. The majority of these DAMPs lead to a decrease in intracellular K^+^ ions, triggering a conformational change in the inactive NLRP3 structure. This conformational change enables NLRP3 to bind to different accessory proteins; on one hand, it binds to never in mitosis-related kinase 7 (NEK7), which stabilizes the active conformation, and on the other, it binds to apoptosis-associated speck-like protein containing a caspase activation and recruitment domain (ASC) that forms long oligomeric ASC filaments from NLRP3 active oligomers [1–4].

Pro-caspase-1 binds to CARD domain buds within the ASC filament, leading to its activation. Activated caspase-1 then cleaves and matures interleukin (IL)−1b and IL-18 cytokines [4–6]. Additionally, caspase-1 also cleaves gasdermin D (GSDMD), thus allowing its N-terminal domain to insert and oligomerize in the plasma membrane, forming pores [7, 8]. These pores facilitate the unconventional release of IL-1β and IL-18. Furthermore, GSDMD pores promote the oligomerization of ninjurin-1 (NINJ1), resulting in plasma membrane rupture and pyroptotic lytic cell death [9]. Pyroptosis is a highly inflammatory form of cell death that is associated with various inflammatory diseases, including arthritis, Alzheimer’s disease, and cardiovascular diseases [10].

Gain-of-function mutations in the *NLRP3* gene have been linked to Cryopyrin–associated periodic syndromes (CAPS). In CAPS patients, myeloid cells exhibit constitutive inflammasome activation, leading to inflammatory flares that are clinically managed with IL-1 receptor antagonists, such as Anakinra. Recently, specific NLRP3 inhibitors targeting gain-of-function variants have demonstrated efficacy in pre-clinical models with CAPS patient samples [11–13]. Furthermore, an oral NLRP3 inhibitor was able to control inflammatory flares during an early clinical trial [14]. Active NLRP3 inflammasomes harbouring CAPS-associated variants impact the immunometabolism of myeloid cells, particularly by reducing glycolysis. Although the mitochondrial citric acid cycle (TCA) remains unchanged in CAPS myeloid cells, a reduction in pyruvate availability may limit TCA function [15]. When this happens, the TCA metabolite itaconate reprograms different anti-inflammatory programs in the cell and is upregulated in inflammatory macrophages. It is synthesized from the decarboxylation of cis-aconitate by the enzyme cis-aconitate decarboxylase (ACOD1). Itaconate and its more electrophilic derivate, 4-octyl itaconate (4-OI), have been identified as potential anti-inflammatory molecules [16]. 4-OI has been shown to exert its effects by dicarboxypropylating Cys548 in mouse NLRP3, thus disrupting the interaction of Cys548 with NEK7, and inhibiting inflammasome formation [17]. However, Cys548 is not conserved in the human NLRP3 and there is no other Cys nearby that could account for human NLRP3 dicarboxypropylation such as occurs in the mouse NLRP3, therefore the mechanism by which 4-OI acts on human NLRP3 is not yet known. Furthermore, 4-OI also affects NF-kB activation, caspase-1 activity and GSDMD pore formation. In peripheral blood mononuclear cells (PBMCs) from CAPS patients treated with lipopolysaccharide (LPS), 4-OI reduces IL-1β production [17]. However, due to its impact on NF-kB activation, the precise effect of 4-OI on autoactive CAPS-associated NLRP3 inflammasomes remains unclear. In the present study, we examine the effects of 4-OI on CAPS-associated NLRP3 inflammasomes by dissociating NLRP3 expression from NF-kB activation. Our findings indicate that 4-OI reduces basal activation of CAPS-associated NLRP3 inflammasomes, thereby impairing the release of the constitutively expressed IL-18 and preventing pyroptosis. However, 4-OI also downregulates *Il1b* gene expression, suggesting that it has a multifaceted impact on the inflammasome pathway in CAPS.

## Results

### 4-octyl itaconate inhibits the constitutively active NLRP3 inflammasome with CAPS-associated variants

Previous studies have demonstrated that 4-OI can inhibit NLRP3 in peripheral blood mononuclear cells from patients with CAPS [17]. However, since 4-OI may affect both NLRP3 priming and pro-IL-1β expression induced by LPS, we aimed to investigate the effects of 4-OI on the constitutive activation of CAPS–associated NLRP3 variants independently of LPS priming and IL-1β release. To achieve this, we used immortalized mouse macrophages deficient in *Nlrp3* with a doxycycline–inducible system to control expression of mutated human NLRP3 in homozygosis [15].

**Fig. 1:**
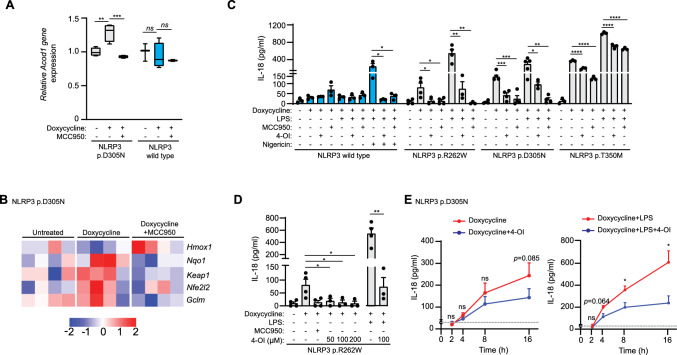
4-octyl itaconate blocks basal IL-18 release from macrophages expressing CAPS-associated NLRP3 variants. **A** Box plot of *Acod1* mRNA expression from *Nlrp3*^−/−^ immortalized macrophages (iMos) treated for 16 h with or without doxycycline (1 µg/ml) to induce the expression of either the human wild type NLRP3 or the p.D305N variant, in the absence or presence of MCC950 (10 µM). The data were represented relative to each untreated condition. **B** Heatmap of gene expression profile of *Nlrp3*^−/−^ iMos treated as in A. Each column represents independent experiments. Gene expression values were normalized using Z-score per row (gene-wise normalization). **C** ELISA for IL-18 released from *Nlrp3*^−/−^ iMos expressing human wild-type NLRP3 or human NLRP3 p.D305N, p.R262W, and p.T350M variants treated as in A, with 4-OI (100 µM). For canonical NLRP3 wild-type activation, iMos were then treated with nigericin (10 µM) 30 min. **D** ELISA for IL-18 released from *Nlrp3*^−/−^ iMos expressing human NLRP3 p.R262W variants induced after treatment for 16 h with doxycycline (1 µg/ml) in the absence/presence of MCC950 (10 µM), 4-OI (50, 100 and 200 µM) or LPS (100 ng/ml). **E** ELISA for IL-18 released from *Nlrp3*^−/−^ iMos expressing human NLRP3 p.D305N variant treated as in C, at different times (0, 2, 4, 8, and 16 h). For **A**–**D**, graphics are representative of *n* = 3–5 independent experiments; For A, the middle line depicts the mean, error bars represent minimum and maximum, and bounds of box represent the 25 th to 75 th percentile respectively; for **C**–**E**, the data are represented as mean ± SEM; For **A**, **B**, data is from GEO dataset #GSE246713. An ordinary one-way ANOVA test was used for **A**–**D**; *t* test was used in E; *p < 0.05; **p < 0.0021; ***p < 0.0002; ****p < 0.0001; *ns*, no significant difference (p > 0.05)

Doxycycline treatment in immortalized mouse macrophages led to the expression of the CAPS-associated variant of human NLRP3 p.D305N, which in turn increased *Acod1* gene expression (Fig. 1A). This increase was not present when wild type NLRP3 was expressed, or when NLRP3 p.D305N was blocked with the specific NLRP3 inhibitor, namely the sulfonyl urea compound named MCC950 (aka. CP-456,773 or CRID3) (Fig. 1A). Additionally, anti-inflammatory itaconate–induced genes related to Nrf2 pathway activation were upregulated in macrophages expressing NLRP3 p.D305N (Fig. 1B), suggesting that itaconate could help limit inflammation during the constitutive activation of NLRP3 with CAPS-associated variants.

To assess the effect of itaconate on the constitutive activation of NLRP3 inflammasome with CAPS-associated variants we used its derivative 4-OI in immortalized mouse macrophages. Treatment with 4-OI significantly reduced IL-18 release upon expression of NLRP3 with the CAPS–associated variants p.R262W, p.D305N and p.T350M (Fig. 1C, Supplementary Fig. 1 A). As expected, wild-type NLRP3 did not induce constitutive basal release of IL-18; however, the canonical activation of NLRP3 did induce IL-18 release, which was then effectively blocked by 4-OI (Fig. 1C). Additionally, 4-OI reduced LPS-induced IL-18 release to levels comparable to the effect of MCC950 (Fig. 1C). Notably, the NLRP3 p.T350M variant induced higher IL-18 release than the other two tested variants, and 4-OI exhibited less efficacy against the p.T350M variant (Fig. 1C), suggesting that in our experimental set-up the p.T350M variant confers a higher degree of inflammasome formation which could be related to the inhibitory effects of 4-OI across the different NLRP3 variants. Different studies assessing p.R262 W, p.D305N and p.T350M NLRP3 variants have found differences in the relative inflammasome activity and clinical manifestations of each of them; therefore, their activity will depend on the particular model and conditions used, although in all cases pathogenic variants present higher inflammasome function than wild type NLRP3 [15, 18–22]. Furthermore, we observed that different concentrations of 4-OI present a similar inhibitory effect on the constitutive IL-18 release induced by the NLRP3 p.R262W variant (Fig. 1D), and a time-dependent inhibition when the NLRP3 p.D305N variant was expressed (Fig. 1E).

**Fig. 2:**
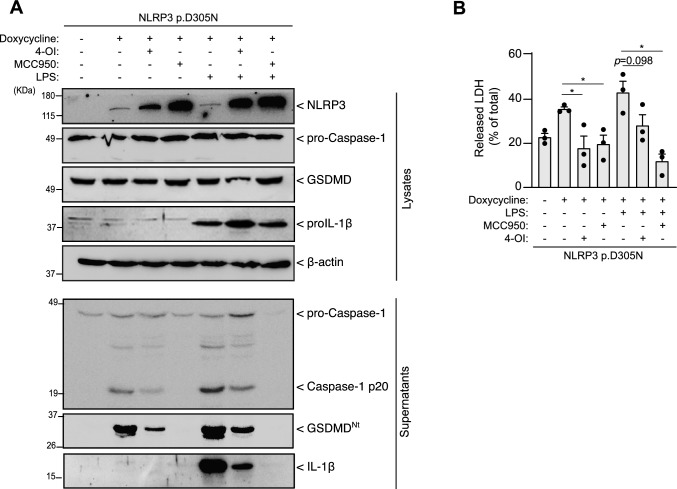
4-octyl itaconate inhibits basal caspase-1 activation, GSDMD cleavage and pyroptosis. **A** Western blot for NLRP3, GSDMD, IL-1β, caspase 1 and β-actin in cell lysates and supernatants from *Nlrp3*^−/−^ immortalized macrophages expressing human NLRP3 p.D305N variant treated with doxycycline (1 µg/ml) in the absence/presence of MCC950 (10 µM), 4-OI (100 µM) or LPS (100 ng/ml) for 16 h. **B** Lactate dehydrogenase (LDH) released from cells treated as in A. Graphic is representative of *n* = 3 independent experiments and data are shown as mean ± SEM; Western blots are representative of *n* = 3 independent experiments; an ordinary one-way ANOVA test was used in B; *p < 0.05; *ns*, no significant difference (p > 0.05)

When the NLRP3 p.D305N variant was expressed, treatment with 4-OI reduced caspase-1 activation, GSDMD processing and pyroptosis (Fig. 2A, B). Notably, mature IL-1β release was reduced by 4-OI only when LPS was added to macrophages expressing the CAPS-associated variant p.D305N (Fig. 2A). Interestingly, using either 4-OI or MCC950 to block NLRP3 inflammasome activation, downstream GSDMD processing and pyroptosis resulted in an increase in intracellular p.D305N NLRP3 variant levels when doxycycline was added (Fig. 2A). These findings show that 4-OI effectively inhibits constitutive NLRP3 inflammasome activation in cells harbouring CAPS-associated variants.

### 4-OI decreases expression of *Tnf*, *Il6* and *Il1b* genes, but not expression of* Il18*

**Fig. 3:**
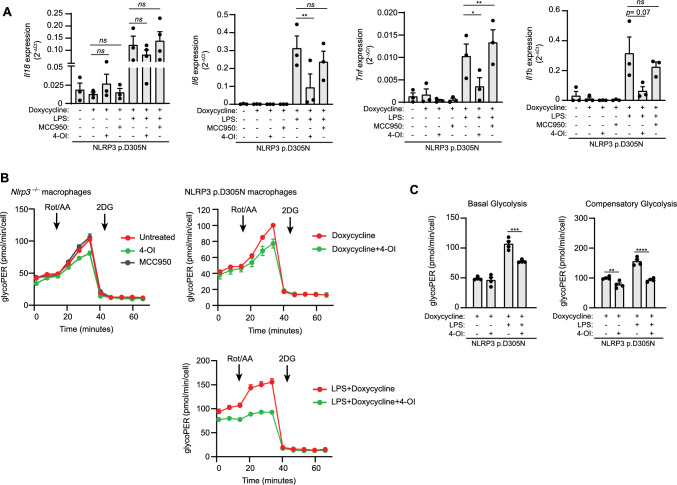
4-octyl itaconate affect the expression of *Il1b*, *Tnf* and *Il6*, but not the expression of *Il18*. **A** Gene expression (2^−ΔCt^) for *Tnf*, *Il1b*, *Il18* and *Il6* from *Nlrp3*^−/−^ immortalized macrophages (iMos) expressing human NLRP3 p.D305N variant induced after treatment for 16 h with doxycycline (1 μg/ml) in the absence/presence of MCC950 (10 µM), 4-OI (100 µM) or LPS (100 ng/ml). **B**, **C** Seahorse analysis of glycolysis in *Nlrp3*^−/−^ iMos treated for 16 h with or without doxycycline (1 µg/ml) to induce the expression of the human NLRP3 p.D305N variant treated as in **A**. For **A** data are representative of *n* = 3 independent experiments. For **B**, **C** data are derived from *n* = 4 biological replicates, which are representative of *n* = 3 independent experiments. Graphics are represented as mean ± SEM; an ordinary one-way ANOVA test was used; *p < 0.05; **p < 0.0021; *ns*, no significant difference (p > 0.05)

We next investigated the impact of 4-OI on LPS-induced cytokine expression in macrophages expressing CAPS-associated NLRP3 variants. As expected, the macrophages presented a constitutive expression of the cytokine IL-18, which remained unaffected by 4-OI treatment (Fig. 3A). Since IL-18 secretion is usually reduced by 4-OI (Fig. 1C), this contrasting effect could be because inflammasome assembly was inhibited independently of macrophage priming. However, when treated with LPS, macrophages expressing the CAPS-associated NLRP3 variant p.D305N showed reduced gene expression levels of *Tnf, Il6* and *Il1b* when treated with 4-OI, whereas *Il18* expression remained unchanged (Fig. 3A). We used MCC950 treatment as a control and found that it did not alter cytokine gene expression (Fig. 3A).

In a separate study, macrophage activation was found to increase glycolysis and thus promote proinflammatory cytokine production [23]; it was then found that 4-OI treatment was able to reduce macrophage glycolysis and impair IL-1β [23]. As described in our previous study [15], macrophages expressing the p.D305N NLRP3 mutant variant also present decreased glycolysis. Therefore, we further investigated the effect of 4-OI on the glycolysis of macrophages that express NLRP3 pathogenic variants. We observed a reduction in glycolysis in 4-OI treated cells, regardless of LPS treatment (Fig. 3B, C). However, 4-OI affected basal glycolysis only in LPS-primed macrophages (Fig. 3B, C). As expected, this reduction was independent of the NLRP3 p.D305N variant, because we found that 4-OI also reduced glycolysis in *Nlrp3*^–/–^ macrophages, whereas MCC950 had no effect on glycolysis in these same macrophages (Fig. 3B). This means that the decrease in glycolysis caused by 4-OI will limit *Il1b* gene expression in macrophages, independently of whether or not these macrophages express the NLRP3 CAPS variant.

These findings suggest that 4-OI decreases cytokine production in LPS-treated macrophages; consequently, 4-OI seems to affect the release of IL-1β in macrophages expressing NLRP3 CAPS variants because it decreases both the expression of *Il1b* gene and the activity of the pathogenic NLRP3 inflammasome.

### 4-OI blocks puncta distribution of NLRP3 p.D305N and formation of ASC-oligomers

**Fig. 4:**
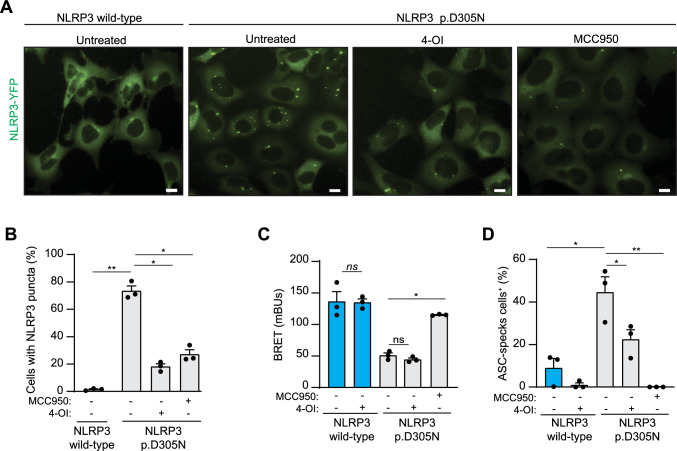
4-octyl itaconate inhibits the ASC-oligomerization induced by NLRP3 p.D305N variant. **A** Representative fluorescence images of HEK293T cells stably expressing wild-type or p.D305N YFP-NLRP3 treated for 24 h with MCC950 (10 µM) or 4-OI (100 µM); NLRP3 is shown in green. Images are representative of *n* = 3 independent experiments. **B** Percentage of HEK293T cells with a puncta distribution of cells treated as in A. **C** BRET signal recorded from HEK293T cells expressing wild-type or p.D305N YFP-NLRP3-Luc, treated for 16 h with MCC950 (10 µM) or 4-OI (100 µM). **D** Percentage of ASC specking HEK293T cells transfected with ASC-RFP and NLRP3-YFP wild type (WT) or p.D305N, treated for 16 h with MCC950 (10 µM) or 4-OI (100 µM). Graphics are representative of at least *n* = 3 independent experiments and data are represented as mean ± SEM; an ordinary one-way ANOVA test was used in **B**, **C**, *t* test was used in **D**; *p < 0.05; **p < 0.0021; *ns*, no significant difference (p > 0.05)

To investigate the impact of 4-OI on inflammasome formation from pathogenic NLRP3 variants, we utilized HEK239T cells that constitutively expressed either wild-type or p.D305N YFP-NLRP3. As previously reported [20], cells expressing the NLRP3 p.D305N variant exhibited a punctate distribution compared to cells expressing wild-type NLRP3 (Fig. 4A). Treatment with 4-OI or MCC950 reduced the percentage of cells with NLRP3 p.D305N puncta (Fig. 4A, B). Interestingly, unlike MCC950, 4-OI did not affect the bioluminescence resonance energy transfer (BRET) signal of either NLRP3 wild-type or p.D305N variant expressed in HEK293T cells (Fig. 4C, Supplementary Fig. 1B), suggesting that 4-OI does not impact NLRP3 structure in the same way that MCC950 does [20]. Furthermore, we used 4-OI to treat HEK293T cells expressing either wild-type or p.D305N YFP-NLRP3 and ASC-RFP. Notably, before treatment, there was a higher percentage of cells with ASC oligomers among cells expressing the NLRP3 p.D305N variant than there was among cells expressing wild-type NLRP3 (Fig. 4D, Supplementary Fig. 1 C). However, after 4-OI treatment, the percentage of cells expressing NLRP3 p.D305N with ASC oligomers decreased (Fig. 4D), indicating that 4-OI affects ASC oligomerization induced by NLRP3 with CAPS variants. Overall, our findings demonstrate that 4-OI modulates the basal activation of NLRP3 with gain-of-function variants in CAPS patients and thus inhibits basal puncta distribution of NLRP3, ASC oligomerization, caspase-1 activation, GSDMD processing and IL-18 release.

## Discussion

We found that 4-OI inhibits inflammasome basal activation from p.D305N CAPS-associated NLRP3 variant by reducing ASC oligomerization, caspase-1 activation, GSDMD processing and IL-18 release. These effects are independent of LPS-priming and not influenced by 4-OI’s potential interference with NF-kB activation. Notably, 4-OI reduced *Il1b* gene expression without affecting *Il18* expression. The inflammasome inhibition by 4-OI was also confirmed in other two pathogenic NLRP3 variants, p.R262W and p.T350M, which together with the p.D305N variant, cover the clinical spectra of CAPS. However, given the heterogeneous response of different CAPS-associated NLRP3 variants when assessed in recombinant systems [21, 22], one limitation of our study is that most of the results were obtained from the p.D305N variant and that therefore the study does not represent all pathogenic NLRP3 variants. That said, the p.D305N variant is one of the strongest NLRP3 variants associated with severe clinical manifestations of CAPS and presents a frequent mutation described in many different CAPS series (i.e. [18, 24–26]). Another limitation is that the study was conducted in homozygotic conditions in which human NLRP3 with CAPS-associated variants were expressed in mouse immortalized macrophages; whereas if there were heterozygous conditions with human monocytes or macrophages, NLRP3 pathogenic variants might react differently to 4-OI. However, previous results show that 4-OI can inhibit NLRP3 in peripheral blood mononuclear cells from patients with CAPS [17] and that similar inflammasome function has been found among recombinant systems expressing NLRP3 pathogenic variants in homozygosis or heterozygosis [15, 21].

NLRP3 activation requires both transcriptional and post-transcriptional priming. LPS is a strong and commonly used priming stimulus for NLRP3 because it induces *Il1b* expression and subsequent inflammasome-dependent IL-1β release, a hallmark of NLRP3 activation. While wild-type NLRP3 needs a second stimulus in order to be activated, CAPS-associated variants of NLRP3 only need stimulus with LPS to induce IL-1β release. NLRP3 inhibitors are typically tested after priming and during the second stimulation, but for mutant NLRP3, inhibitors are co-administered with the priming agent to measure IL-1β release. In this context, in addition to NLRP3 activation, 4-OI could target both LPS-induced priming and *Il1b* expression. Recent findings indicate that there is a series of inflammasome-formations for NLRP3 with different pathogenic mutations, ranging from the autoactivation of a functional inflammasome without cellular priming to mutations that do require priming [15, 21, 22]. Notably, in all three NLRP3 variants tested in our study model (p.R262W, p.D305N and p.T350M) we found that IL-18 was constitutively released without cellular priming and that 4-OI reduces mutant NLRP3 inflammasome activation even in the absence of priming. Previous data suggest that itaconate and its derivate 4-OI promote dicarboxypropylation of Cys548 in mouse NLRP3, disrupting its interaction with NEK7 [17, 27]. However, there is no homologue to Cys548 in the human NLRP3 sequence, raising the question of the potential dicarboxypropylation of 4-OI in human NLRP3. Although we have not directly analyzed the interaction between mutant human NLRP3 and NEK7, our observations show decreased puncta distribution of NLRP3 p.D305N and a decrease in the formation of ASC oligomers, without any effect on the active conformation of NLRP3 auto-active p.D305N variant. Interestingly, the NLRP3 inhibitor MCC950 stabilizes the inactive structure of mutant NLRP3 [3, 20]. Therefore, distinct mechanisms are likely to underlie the different actions of MCC950 and 4-OI in inhibiting mutant NLRP3 inflammasome activation.

Additionally, previous studies have shown that itaconate induces several anti-inflammatory responses, including the activation of Nrf2 [28]. In our study, we found that macrophages expressing pathogenic variants associated with CAPS increase the expression of the *Acod1* gene and upregulate genes related to the Nrf2 pathway, suggesting that itaconate could be regulating sterile inflammatory flares in the constitutive activation of p.D305N NLRP3 inflammasome.

Different studies have shown that 4-OI reduces NF-kB activation, leading to decreased production of pro-inflammatory cytokines [23, 28–32]. In macrophages expressing mutant NLRP3, we found that after LPS treatment 4-OI downregulates *Il1b*, *Il6*, and *Tnf* gene expression. Notably, basal or LPS-induced *Il18* expression remains unaffected by 4-OI. Additionally, itaconate inhibits both priming and activation of the canonical NLRP3 inflammasome in macrophages [30]. Itaconate also confers tolerance to late NLRP3 inflammasome activation through prolonged LPS priming [33]. Furthermore, itaconate acts downstream of inflammasome-induced ASC oligomerization, thus impacting caspase-1 activation and GSDMD pore formation, with GSDMD being itaconated on Cys77 [33].

Moreover, several studies have highlighted the importance of glycolysis in the production of IL-1β and the development of inflammation. 4-OI inhibits several glycolytic enzymes, which in turn reduces IL-1β [23, 34]. Consistent with this, we found that 4-OI reduces glycolysis in macrophages expressing or not the pathogenic p.D305N NLRP3 variant, which could potentially contribute to limiting IL-1β production during sterile inflammatory flares in CAPS patients. Therefore, the effect of 4-OI on IL-1β release could (*i*) affect *Il1b* induction by reducing glycolysis, and (*ii*) block constitutively active NLRP3 p.D305N inflammasome associated with CAPS and thus impair IL-1β maturation. Since NLRP3 inflammasome activity in pathogenic variants also limits *Il1b* expression through glycolysis reduction [15], direct inhibition of the inflammasome through treatment with MCC950 would restore glycolysis. Then, 4-OI and MCC950, both of which have different acting mechanisms for inhibiting the NLRP3 inflammasome, would have the opposite effect on glycolysis ([15] and this study).

Therefore, 4-OI affects several inflammatory pathways and induces anti-inflammatory signalling cascades [23, 28, 31, 34], and it is not specific for the NLRP3 inflammasome. Numerous studies have shown that 4-OI induces Nrf2 pathway, thus reducing the inflammation. This has been observed in ex vivo and preclinical models of different diseases [28, 35–37]. In addition, some preclinical models have shown 4-OI to have anti-inflammatory actions independent of Nrf2 pathway, such as direct modification of JAK1 [38] and also modification of STING, thus inhibiting its phosphorylation [39]. Other studies have found that 4-OI acts as an anticoagulant that inhibits tissue factor-mediated coagulopathy [40]. These different targeted pathways need to be analysed in greater depth in vivo and in early clinical trials of 4-OI, as this tight balance of affected pathways could have a beneficial anti-inflammatory effect but also could affect host metabolism. Overall, our study demonstrates that therapeutic molecules based on itaconate could have positive therapeutical implications for CAPS with p.R262W, p.D305 N and p.T350M variants that control inflammatory flares acting on different levels, namely by reducing *Il1b* expression and preventing mutant NLRP3 autoactivation.

## Methods

*Reagents*. Nigericin, doxycycline, 4-octyl itaconate and MCC950 (CP-456773) were from Sigma-Aldrich. Ultrapure *E. coli* LPS serotype 0111:B4 (tlrl-3pelps) was from InvivoGen.

*Immortalized mouse macrophages*. In this study we used *Nlrp3*^−/−^ immortalized mouse macrophages (a gift from I. Hafner-Bratkovič, National Institute of Chemistry, Ljubljana, Slovenia) with a doxycycline-inducible system for the expression of human NLRP3-YFP wild-type or p.D305N, p.T350M and p.R262W variants as previously described [3, 15, 20]. Note that in this study we used annotation of the amino acid according to the NLRP3 sequence ID# AAI43360 (GenBank, NCBI), which starts two amino acids before the alternative NLRP3 sequence ID# NM_001243133 (GenBank, NCBI). Therefore, the p.D305N, p.T350M and p.R262W nomenclature used in this study correspond to p.D305N, p.T348M and p.R262W respectively. Cells were treated with doxycycline (1 µg/ml) in the presence or absence of 4-OI, MCC950 or LPS. Concentrations and incubation times with these compounds are indicated in the figure legends.

*Western blot*. The protocol followed has been previously described [15]. In brief, stimulated cells were centrifuged at 13,000 x*g* for 10 min at 4 °C and then lysed on ice for 30 min in a buffer composed of 50 mM Tris–HCl pH 8.0, 150 mM NaCl, 2% Triton X-100 and 100 μl/ml of protease inhibitor mixture (Sigma-Aldrich). Cell-free supernatants were obtained by centrifugation at 600 x*g* for 5 min at 4 °C and then concentrated using a 10 kDa cut-off column (UFC501024, Merk-Millipore). Lysates and supernatants were diluted in Laemmli buffer (Sigma-Aldrich) and incubated at 95ºC for 5 min. Electrophoresis was performed in a 12% precast Criterion polyacrylamide gel (Biorad). Then the gels were transferred to nitrocellulose membranes by electroblotting using the Trans-Blot Turbo transfer system (Biorad). Membranes were blocked with skimmed milk 5% (v/v) and hybridized for 16 h at 4ºC with the following antibodies: anti-Caspase-1 (p20) clone Casper-1 (AG-20B-0042, Adipogen, 1:1000), anti-NLRP3 clone Cryo-2 (AG-20B-0014, Adipogen, 1:1000), anti-GSDMD clone EPR19828 (ab209845, Abcam, 1:5000), anti–IL-1β clone H-153 (sc-7884; 1:1000), or horseradish peroxidase (HRP)-anti-β-actin clone C4 (sc-47778HRP, Santa Cruz, 1:10,000). After that, membranes were washed three times and incubated with the appropriate secondary antibodies: horseradish peroxidase anti-IgG rabbit (NA9340 V, 1:5000) or horseradish peroxidase anti-IgG mouse (NA9341 V, 1:5000) (both from Cytiva). Membranes were revealed using ECL Plus reagent (Cytiva) and a ChemiDoc Imaging System (BioRad).

*Lactate dehydrogenase determination*. To determinate pyroptotic cell death, we used the Cytotoxicity Detection kit (Roche) on cell supernatants according to manufacturer instructions as previously described [15]. The assay was detected at 492 nm with a correction at 620 nm using a Synergy Mx (BioTek) plate reader.

*ELISA*. IL-18 was detected in cell supernatants using the mouse IL-18 ELISA kit (BMS618-3, Invitrogen; and DY7625-05, R&D systems) following manufacturer instructions and detected at 450 nm with 540 nm correction using a Synergy Mx (BioTek) plate reader.

*Quantitative reverse transcriptase-PCR analysis*. The method described in [15] was followed. In brief, the RNeasy kit (Qiagen) was used to extract total RNA. RNA was quantified in a NanoDrop 2000 (Thermo Fisher) and reverse transcripted with the BioRad kit iScript™ cDNA Synthesis according to manufacturer instructions. A CFX96™ Real-time System (BioRad) with SYBR Green mix (Takara) and predesigned KiCqStart primers for mouse *Il1b, Tnfa, Il18,* and *Il6* (Sigma-Aldrich) were used for quantitative PCR. Gene expression was normalized to 18S.

*Transcriptome analysis*. Data analyzed in this study is from the GEO database with accession number GSE246713 [15].

*Cells and transfections*. HEK293T cells (CRL-11268, ATCC) were cultured at 37 ºC, with 5% CO_2_ in a humidified incubator with DMEM/F-12 media (Lonza) supplemented with 10% fetal calf serum (Life Technologies), 1% penicillin–streptomycin (Life Technologies) and 2 mM GlutaMAX (Life Technologies). For cell transfection, Lipofectamine 2000 was used according to the manufacturer’s instructions. The plasmids transfected encoded for ASC-RFP, YFP-NLRP3-Luc or YFP-D305N-NLRP3-Luc.

*Flow cytometry*. Intracellular ASC-speck formation was evaluated by seeding 7 × 10^5^ HEK293T cells stably expressing wild-type or p.D305N YFP-NLRP3, which were then transfected with ASC-RFP. MCC950 and 4-OI were added to the cells 2 h post-transfection. ASC specks were detected 24 h post-transfection through Time-of-Flight Inflammasome Evaluation [41] and analysed by flow cytometry using LSRFortessa (BD Biosciences) and the FCS express software (De Novo Software).

*Bioluminescence resonance energy transfer*. Opaque white 96-well plates coated with poly-L-lysine were used to plate HEK293T cells expressing wild-type or p.D305N NLRP3 BRET sensors [20]. Cells were treated with MCC950 (10 µM) or 4-OI (100 µM) for 16 h at 37ºC and 5% CO_2_. After incubation, cells were washed and treated with MCC950 or 4-OI during BRET determination. Light signal was recorded after 5 min of 5 μM coelenterazine-H addition and was detected with the following filters: 485 ± 20 nm (Renilla luciferase (Luc) filter) and 530 ± 25 nm (YFP filter) at 37 °C. A Synergy Mx (BioTek) plate reader was used to capture light. The BRET ratio was calculated as the emission ratio 530 nm/485 nm of the BRET sensor minus the ratio of the Luc only tagged NLRP3. Results were expressed in milliBRET units (mBU).

*Fluorescence microscopy*. HEK293T cells constitutively expressing NLRP3 wild-type or p.D305N variant both tagged with YFP (5 × 10^5^ cells per well of a 6 well-plate) were treated for 24 h with 100 µM 4-OI or 10 µM MCC950 and imaged with a Nikon Eclipse *Ti* microscope (20 × S Plan Fluor objective with numerical aperture 0.45) and 472 nm/520 nm filter sets (Semrock). Images were acquired with a digital Sight DS-QiMc camera (Nikon) and the NIS-Elements AR software (Nikon). Images were analysed using ImageJ (US National Institutes of Health).

*Seahorse assay*. Immortalized macrophages expressing NLRP3 pathogenic variants were plated in DMEM high glucose medium (Biowest) without FBS in a Seahorse adherent 96-well plate (25.000 cells/well, Agilent Technologies). After 16 h of macrophage treatments (1 µg/ml doxycycline, 100 ng/ml LPS and 10 µM MCC950 or 100 µM 4-OI), cell media were removed and replaced with DMEM seahorse medium supplemented with 5 mM glucose, 1 mM pyruvate and 2 mM glutamine (Agilent Technologies). Cells were incubated for 45 min at 37 °C without CO_2_ and then a glycolytic rate assay kit (Agilent Technologies) was used according to the manufacturer’s instructions. Measurements were performed using an XF96e Analyzer (Agilent Technologies) and, to normalize the number of cells in the calculated OCR and ECAR values, cell nuclei were stained with Hoechst (3 mM, Sigma-Aldrich). Data was recovered using the Wave software version 2.6 (Agilent Technologies).

*Statistics and reproducibility*. GraphPad Prism version 9 (GraphPad Software Inc) was used for statistical analyses. D’Angostino and Pearson omnibus K2 test was used to determine the normality of the data. Outliers were identified by the ROUT method (*Q* = 1%) and were removed from the analysis and representations. All data show the mean value and the error bars represent standard error (SEM). For non-parametric data, a Mann–Whitney U test was used to compare two groups. For parametric data, a *t* test was used to compare two groups. Multiple group comparison was analysed using the Kruskal–Wallis test if the data was non-parametric or the ANOVA test if the data was parametric. *p* values are indicated with asterisks in the figures and the ranges are noted in the figure legends, with *p* > 0.05 not being considered significant (*ns*).

## Supplementary Information

Below is the link to the electronic supplementary material.

**Supplementary Figure 1. Expression of NLRP3 with pathogenic variants.** (**A**) Western blot for NLRP3 and β-actin in cell lysates from *Nlrp3*^−/−^ immortalized macrophages (iMos) treated for 16 h with or without doxycycline (1 μg/ml) to induce the expression of either the human wild type NLRP3 or the p.R262W, p.D305N and p.T350M variants. Related to main Figure 1C. (**B**) YFP fluorescence from HEK293T cells expressing wild-type or p.D305N YFP-NLRP3-Luc, treated for 16 h with MCC950 (10 μM) or 4-OI (100 μM). Related to main Figure 4C. Graphics include data of *n* = 3 independent experiments represented as mean ± SEM; an ordinary one-way ANOVA test was used: *ns*, no significant difference (*p* > 0.05). (**C**) Gating strategy to analyse the percentage of ASC specking cells in a gate with low expression (calculated as mean fluorescence intensity, MFI) for NLRP3-YFP wild type (WT) or p.D305N, treated for 16 h with MCC950 (10 μM) or 4-OI (100 μM) as indicated. Related to main Figure 4D

Supplementary file2 (PDF 9039 KB)

Supplementary file3 (XLSX 655 KB)

## Data Availability

Enquiries about data availability should be directed to the authors. Raw data is presented in Supplementary file 3.
