# Supplementary figures and images for: 4-octyl itaconate reduces human NLRP3 inflammasome constitutive activation with the cryopyrin-associated periodic syndrome p.R262W, p.D305N and p.T350M variants
Source: Cell Mol Life Sci. 2025 May 23;82(1):209. doi: 10.1007/s00018-025-05699-5 (PMC12102053; doi:10.1007/s00018-025-05699-5)

A

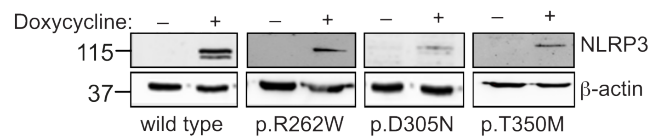

B

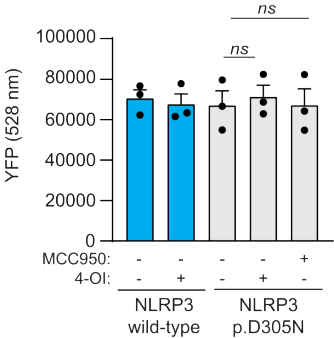

C

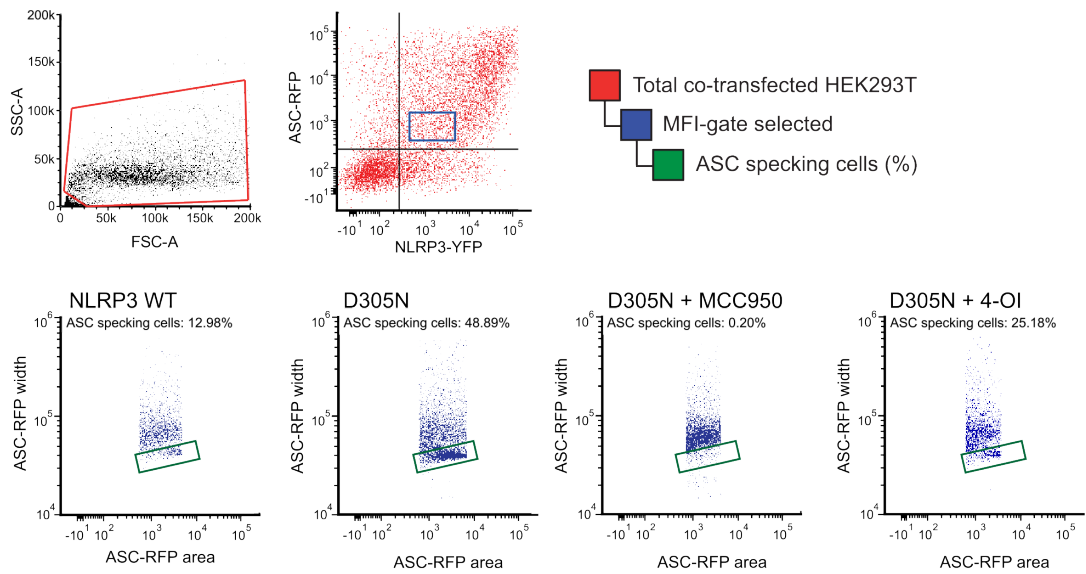

Supplement: Supplementary file 1 — Supplementary Figure 1. Expression of NLRP3 with pathogenic variants. (A) Western blot for NLRP3 and β-actin in cell lysates from Nlrp3−/− immortalized macrophages (iMos) treated for 16 h with or without doxycycline (1 μg/ml) to induce the expression of either the human wild type NLRP3 or the p.R262W, p.D305N and p.T350M variants. Related to main Figure 1C. (B) YFP fluorescence from HEK293T cells expressing wild-type or p.D305N YFP-NLRP3-Luc, treated for 16 h with MCC950 (10 μM) or 4-OI (100 μM). Related to main Figure 4C. Graphics include data of n = 3 independent experiments represented as mean ± SEM; an ordinary one-way ANOVA test was used: ns, no significant difference (p > 0.05). (C) Gating strategy to analyse the percentage of ASC specking cells in a gate with low expression (calculated as mean fluorescence intensity, MFI) for NLRP3-YFP wild type (WT) or p.D305N, treated for 16 h with MCC950 (10 μM) or 4-OI (100 μM) as indicated. Related to main Figure 4D [file 18_2025_5699_MOESM1_ESM.pdf]
