# Supplementary material for: 4-octyl itaconate reduces human NLRP3 inflammasome constitutive activation with the cryopyrin-associated periodic syndrome p.R262W, p.D305N and p.T350M variants
Source: Cell Mol Life Sci. 2025 May 23;82(1):209. doi: 10.1007/s00018-025-05699-5 (PMC12102053; doi:10.1007/s00018-025-05699-5)

Uncropped Western blot of Figure 2A

NLRP3 p.D305N

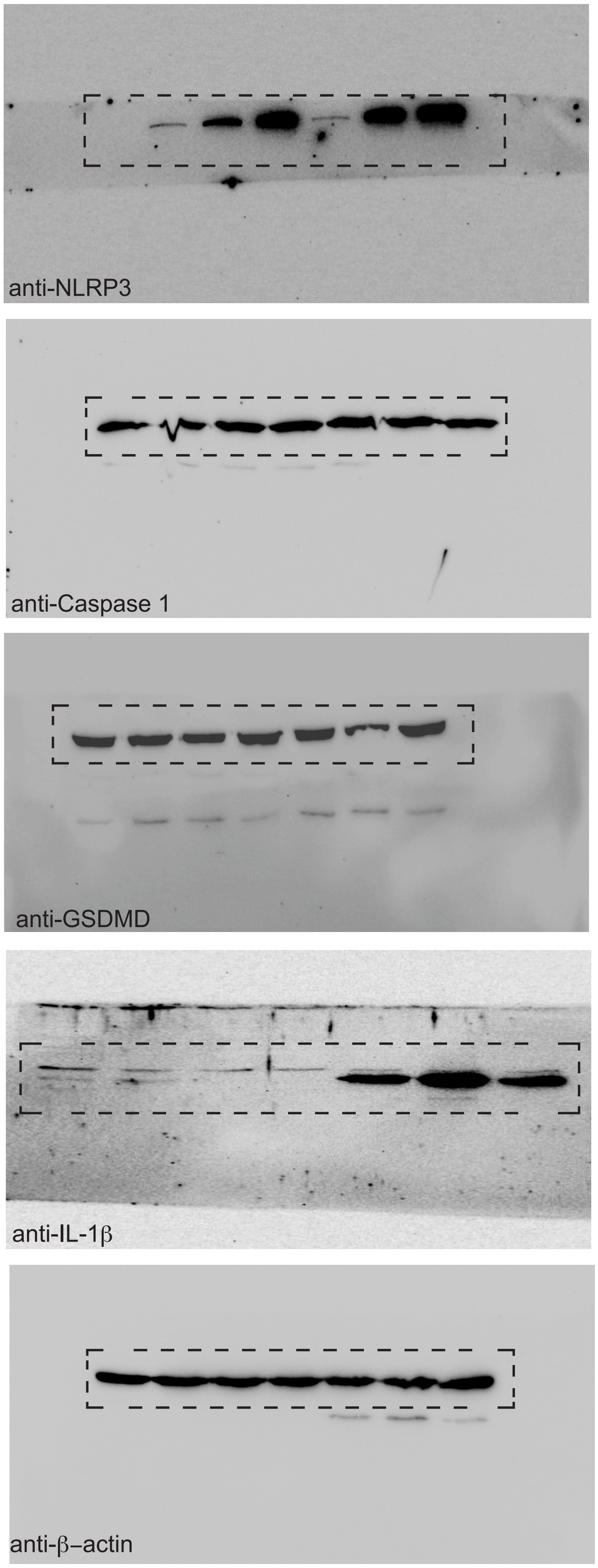

NLRP3 p.D305N

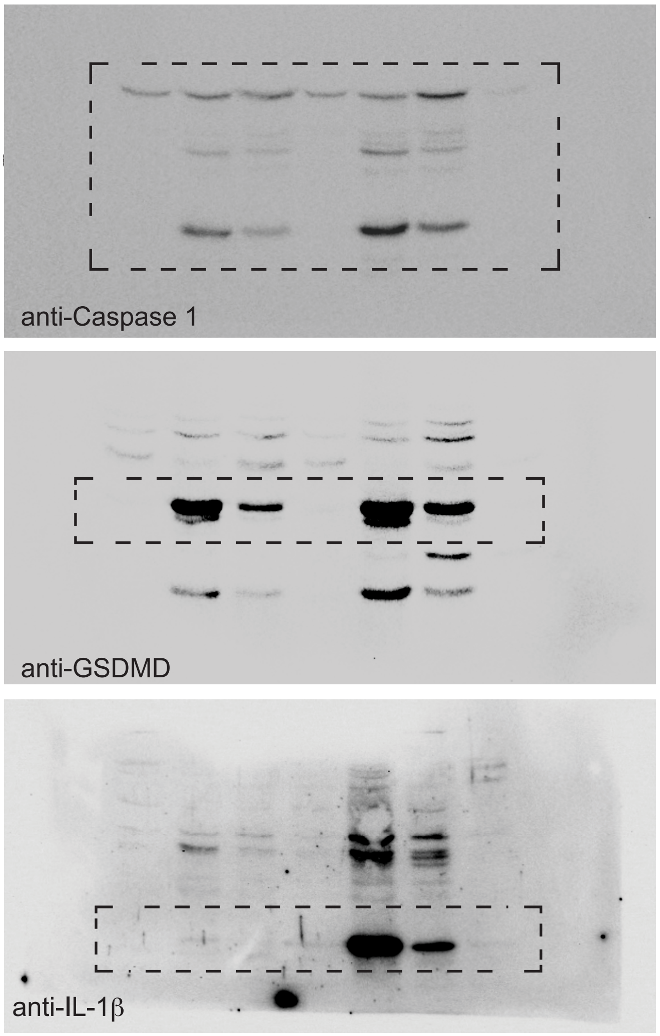

Uncropped Western blot of Supplementary Figure 1A

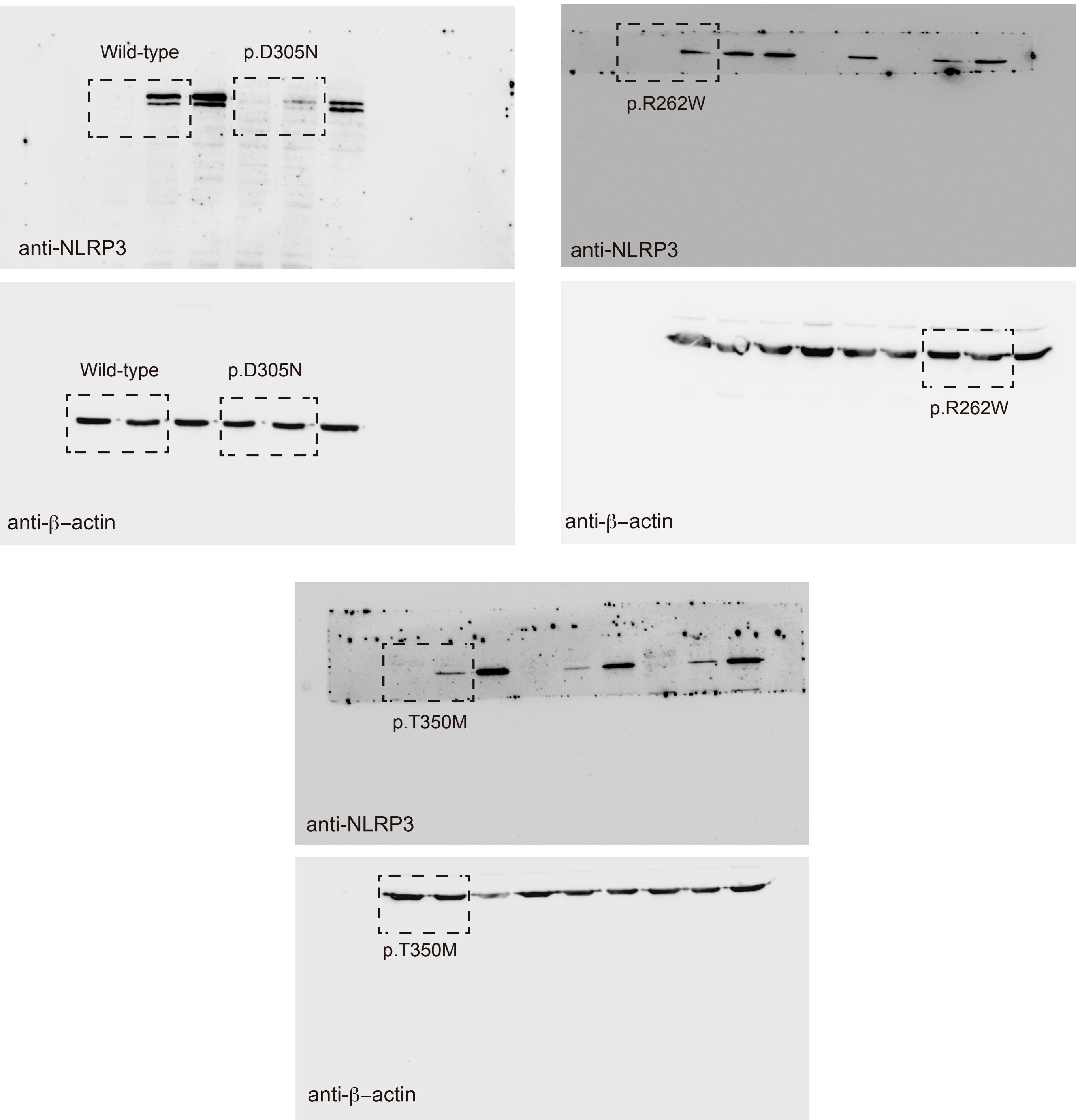

Supplement: Supplementary file 2 — Supplementary file2 (PDF 9039 KB) [file 18_2025_5699_MOESM2_ESM.pdf]
